# Bixin Attenuates High-Fat Diet-Caused Liver Steatosis and Inflammatory Injury through Nrf2/PPAR*α* Signals

**DOI:** 10.1155/2021/6610124

**Published:** 2021-02-02

**Authors:** Shasha Tao, Youjing Yang, Jianzhong Li, Hongyan Wang, Yu Ma

**Affiliations:** ^1^Chongqing University Central Hospital & Chongqing Emergency Medical Center, No. 1 Jiankang Road, Yuzhong District, Chongqing 400014, China; ^2^School of Public Health, Medical College of Soochow University, 199 Ren'ai Road, Suzhou 215123, China; ^3^Department of Nephrology, The First Affiliated Hospital of Soochow University, Suzhou, Jiangsu 215006, China

## Abstract

Nonalcoholic fatty liver disease is the most common liver disease worldwide. Hepatic steatosis and oxidative stress are the main characteristics of NAFLD (nonalcoholic fatty liver disease), which also affect its prognosis. Bixin acts as novel Nrf2 (NF-E2 p45-related factor 2) activator with the cytoprotection against oxidative stress and inflammation; this study mainly focused on the mechanism of Nrf2 activation by bixin and explored its potential feasibilities in long-term high-fat diet- (HFD-) caused hepatic steatosis and inflammatory response *in vitro* and *in vivo*. Bixin was found to activate Nrf2 signals by the modification of critical Keap1 (Kelch-like ECH-associated protein 1) cystine and competitive interaction with Keap1 with upregulating P62 mRNA and protein expression. In human liver cells exposed to FFA (free fatty acid), bixin displayed a pronounced cytoprotective activity with upregulation of Nrf2-mediated gene expression, such as PPAR*α* and its targets related with fatty acid oxidation. In HFD-fed mice, systemic administration of bixin attenuated lipid accumulation, decreased oxidant inflammatory damage in the liver, and reduced circulating lipid levels through Nrf2. Different from most of other established inducers, bixin activated Nrf2 signals through two different mechanisms with safe administration for protection of oxidant inflammatory damage and attenuation of lipid accumulation in the *in vivo* long-term HFD-fed mice. Bixin represents a prototype Nrf2 activator that displays cytoprotective activity upon system administration targeting hepatic steatosis and oxidant inflammation originating from long-term HFD-fed mice. And bixin-based Nrf2-directed systemic intervention may also provide therapeutic benefit in protecting other organs in the process of metabolic syndrome.

## 1. Introduction

Nonalcoholic fatty liver disease (NAFLD) affects approximately 30% of adult population and has become one of the most common liver diseases around the world [1–3]. Characterized by steatosis, inflammation, cell ballooning, tissue necrosis, or apoptosis, NAFLD is regarded as the hepatic manifestation of metabolic syndrome, ranging from simple hepatic steatosis to severe stages of nonalcoholic steatohepatitis (NASH), which could be further developed into cirrhosis and hepatocellular carcinoma [4–6]. Therefore, development of novel therapeutic strategies to limit and prevent the initiation and development of NAFLD is urgently needed.

Lipid accumulation and oxidative stress are considered as the major factors that affect the procedure of NAFLD [7, 8]. Hepatic accumulative lipid induces the tissue oxidative stress, which subsequently causes the lipid peroxidation [9, 10]. These series of events lead to hepatic damage, such as inflammatory response, cell apoptosis, or necrosis, which exacerbate the NAFLD. Studies have reported that levels of reactive oxygen species (ROS) in NAFLD patients are markedly increased compared with those in healthy subjects [11, 12]. Thus, attenuation of lipid accumulation and suppression of oxidative stress would be an efficient method to treat the NAFLD. Cumulative studies reported that the NF-E2 p45-related factor 2 (Nrf2) signals serve as a critical cellular defense system that prevents tissue damage in the process of several diseases by regulating a range of genes [13–15]. We and others have also demonstrated the feasibilities of diet-derived Nrf2 activators including sulforaphane (SF), cinnamaldehyde (CA), and tanshinone I (T-I) for the prevention of tissue damage in various diseases (including inflammation, fibrosis, diabetic nephropathy, and tumor) through modulation of the Nrf2-dependent cellular defense mechanism [16–18]. Besides that, Nrf2 signals are also involved in negatively controlling the lipid accumulation not only by suppressing the FFA uptake factors such as cluster of differentiation 36 (CD36) and fatty acid transport proteins (FATPs) but also through regulating fatty acid metabolism and transport by activating peroxisome proliferator-activated receptor *α* (PPAR*α*) signals [19, 20]. For example, depletion of n-3 long polyunsaturated fatty acids decreased PPAR*α* signals, contributing liver steatosis by inhibiting FFA oxidation [21].

Apocarotenoid bixin is a Food and Drug Administration- (FDA-) approved natural food colorant and additive, which is extracted from the seeds of the *achiote* tree and proven to be safe for human administration [22]. Derived from lycopene through oxidative cleavage, bixin has traditionally been used in Mexico and South America to treat infectious and inflammatory diseases like skin, prostate, and chest pain [23, 24]. Previous in vitro biochemical measurement indicated bixin could quench the environmental reactive oxygen species (ROS). Similarly, animal studies also showed that bixin protects against oxidative DNA damage and suppresses lipid peroxidation [25]. Furthermore, our previous study has identified that bixin is a novel Nrf2 inducer, which could quench the ROS and inhibit the lung tissue inflammation and fibrosis [26, 27]. In addition, we also found that bixin could protect against UV exposure-caused skin tissue damage in an Nrf2-dependent manner as well [28].

Nrf2 is primarily regulated by Keap1, a substrate adaptor for a Cul3-containing E3 ubiquitin ligase [29]. Under basal conditions, the Keap1-Cul3-E3 ubiquitin ligase complex constantly ubiquitinates Nrf2 protein and promotes it for degradation by 26s proteasome to maintain it at a low level [30]. Nrf2 is primarily localized in a complex with Keap1 via direct protein-protein interactions between the Keap1 Kelch domain and the ETGE (strong binding) and DLG (weak binding) motifs of Nrf2 Neh2 domain [31]. So far, there are two potential mechanisms reported to activate Nrf2 signals via regulation of Keap1: canonical mechanism, which confers the activation by cellular exposure to oxidative or electrophilic stress that modified the critical cysteine residues in Keap1, leading to a conformational change of Keap1-Cul3-E3 complex that releases the bind with DLG motif and subsequently stabilized Nrf2 [32], while the noncanonical mechanism does not modify Keap1 cysteines. P62 (also termed as sequestosome 1, SQSTM1) is an important mediator that involved in the noncanonical mechanism, which binds with the Kelch domain of Keap1 with its pSTGE motif [33]. By this competition, Nrf2 was released and translocated to the nucleus to activate its target genes.

In this study, we explored the mechanism of bixin in the activation of Nrf2 signals and demonstrated that activation of Nrf2 by bixin suppressed the NF-*κ*B pathway and upregulated the PPAR*α* with its targets, which plays a pivotal role in hepatic steatosis and inflammation by using a high-fat diet- (HFD-) fed mice model. These results suggest that pharmacological activation of Nrf2 by bixin may provide therapeutic benefit against metabolic syndrome-related organs' abnormal and oxidant inflammatory damage.

## 2. Materials and Methods

### 2.1. Chemicals, Antibodies, and Cell Culture

Bixin was purchased from Spectrum (New Brunswick, NJ). Oleic acid, palmitic acid, DAPI, cycloheximide, rapamycin, and bafilomycin A1 were purchased from Sigma (St. Louis, MO). MG132 was purchased from Amquar (Colorado, USA). Primary antibodies against Nrf2 (sc-13032), Keap1 (sc-15246), HO-1 (sc-136960), P65 (sc-8008), p-P65 (sc-136548), P62 (sc-28359), ubiquitin (Ub) (sc-8017), PPAR*α* (sc-398394), Acox1 (sc-517306), CPTII (sc-37294), and GAPDH (sc-32233) were purchased from Santa Cruz (Santa Cruz, CA). Antibody against 8-oxo-dG was purchased from Trevigen (Gaithersburg, MD; #3154-MC-050). Horseradish peroxidase- (HRP-) conjugated secondary antibodies were all purchased from ImmunoWay (Plano, TX; #RS001, #RS002). Alexa Fluor 488 anti-mouse and Alexa Fluor 594 anti-rabbit were purchased from Santa Cruz. Human hepatic cell line LO2 was purchased from Cell Bank of the Chinese Academy of Sciences in Shanghai, China. Cells were cultured in Dulbecco's modified Eagle's medium (DMEM) containing 10% FBS, 100 units/ml penicillin, and 100 *μ*g/ml streptomycin. All cells were kept in a humidified incubator at 37°C with 5% CO_2_.

### 2.2. Transfections of siRNA and Plasmids, and Luciferase Reporter Gene Assay

Plasmid transfection was performed with Lipofectamine 2000 (Invitrogen), and HiPerfect transfection reagent (Qiagen, Hilden, Germany) was used for transfection with small interfering RNA (siRNA) according to the manufacturer's instructions. Nontargeted siRNA (Ctrl-siRNA, #1027281), Nrf2-targeted siRNA (Nrf2-siRNA, #SI00657937), and P62-targeted siRNA (P62-siRNA, #SI00057596) were purchased from Qiagen. Activation of Nrf2 transcriptional activity was performed as previously described [34]. LO2 cells were cotransfected with expression vectors for either Keap1 wild-type (Keap1 WT) or a mutant Keap1 (Keap1 C151S), along with mGst-ARE firefly and Renilla luciferase reporters. At 24 h posttransfection, cells were treated with SF (5 *μ*M), tBHQ (50 *μ*M), As (5 *μ*M), or bixin (40 *μ*M) for 16 h, and then lysed for analysis of the reporter gene activity using the Beyotime Biotechnology dual-luciferase reporter gene assay system.

### 2.3. Cell Viability Assay

Potential cytotoxicity of bixin in LO2 cells was assessed by the functional impairment of the mitochondria with 3-(4,5-dimethylthiazol-2-yl)-2,5-diphenyltetrazolium bromide (MTT, Sigma). About 1 × 10^4^ cells per well were plated in a 96-well plate. After overnight incubation, the cells were treated with different doses of bixin for 48 h. Then, 40 *μ*g MTT was added into the cells. After 2 h incubation, the medium was removed by aspiration. 100 *μ*l isopropanol/HCl was added into each well to dissolve the crystals. Absorbance at 570 nm was measured by a synergy 2 multimode microplate reader (BioTek, Seattle, USA).

### 2.4. ROS and Cell Apoptosis Detection

Followed by Ctrl-siRNA or Nrf2-siRNA transfection, cells were treated with bixin 40 *μ*M for 24 h prior to FFA exposure 1 mM (oleic acid/palmitic acid = 2 : 1 dissolved in 20% BSA). In the detection of ROS, the cells were incubated with fresh medium containing 10 *μ*g/ml 2′,7′-dichlorodihydrofluorescein diacetate (H_2_DCFDA, Sigma) for 1 h. And cell apoptosis detection was performed with Annexin V-FITC Apoptosis Detection Kit purchased from Beyotime (China). Finally, the cells were resuspended to 10^6^/ml in PBS. Fluorescence was measured by flow cytometry (Becton Dickinson and Company, USA) with the excitation at 488 nm and emission at 515 nm (ROS detection). The entire process was performed in the dark.

### 2.5. Preparation of Nuclear and Cytoplasmic Fractions

Nuclear and cytoplasmic fractions were prepared according to manufacturer's instructions (CWBiotech, China, CW0199S). Briefly, cells were scraped into ice-cold PBS, centrifuged at 3000*g*, and resuspended in ice-cold buffer A with protease inhibitor cocktail. After added buffer B, the mixture was centrifuged at 13400*g* for 15 min for collecting the cytoplasmic fraction. Then, the buffer C was used to solve the nuclear fraction from the cell pellet. To ensure the subcellular fractions were separated properly, subcellular lysates were verified by the antibodies against the corresponding fractions.

### 2.6. Immunoblot Analysis, Immunoprecipitation, Ubiquitination Assay, Protein Half-Life, Indirect Immunofluorescence, and Oil Red O Staining

The immunoblot analysis was employed to detect protein expression. Cell and tissue lysates were prepared the same as previously reported [35]. Lysates were run in the SDS-polyacrylamide gel and subjected to immunoblot analysis with the indicated antibodies. For immunoprecipitation and ubiquitination assay, cells were harvested in RIPA buffer (Thermo) and incubated with the indicated antibodies (1 *μ*g) together with protein A agarose beads (Invitrogen) at 4°C for 16 h. Immunoprecipitated proteins were analyzed by immunoblot with antibodies against Ub, Keap1, Nrf2, and P62 (Santa Cruz). To clarify Nrf2 stability, cell lysates at different time points from control- or bixin-treated cells were subjected to immunoblot analyses with the anti-Nrf2 and anti-GAPDH antibodies. The intensity of Nrf2 and GAPDH bands was quantified with ImageJ and plotted against the time after addition of cycloheximide (CHX). For indirect immunofluorescence, cells were seeded on round glass coverslips (Fisher Scientific). After fixed with chilled methanol, coverslips were incubated with the primary antibodies and the respective secondary antibodies for 50 min each. Coverslips were mounted with antifade mounting solution (Invitrogen). For Oil Red O staining, the cells' coverslips and frozen liver sections (10 *μ*m) were fixed and stained with freshly made Oil Red O solution. Images were captured with a fluorescence microscope (Leica DM2500).

### 2.7. RNA Extraction and Quantitative Real-Time PCR (qRT-PCR)

Total RNA was extracted from both cells and liver tissues with TRIzol reagent purchased from CWBio (Beijing, China). cDNA was generated with equal amounts of mRNA with HiFiScript cDNA synthesis kit according to the manufacturer's instructions (CWBio, Beijing, China). Primer sequences of human Nrf2, HO-1, NQO1, P62, and GAPDH and mouse P62, Nrf2, HO-1, NQO1, IL-6, TNF-*α*, and GAPDH were described previously [26, 28]. The ABI 7500 (Applied Biosystems) was used to evaluate mRNA expression. Procedures of qRT-PCR were as follows: initial denaturation (95°C, 10 min), 40 cycles of amplification (95°C, 15 s; 60°C, 1 min), and melting curve (95°C, 15 s; 60°C, 1 min; 95°C, 15 s; 60°C, 15 s) with 96-well PCR plates(nest, 402101). Mean crossing point (Cp) values and standard deviations (SD) were determined. Cp values were normalized to the respective Cp values of human GAPDH or mouse *β*-actin reference gene. Data are presented as a fold change in gene expression compared to the control group.

### 2.8. Animals and Treatments

Nrf2 wild-type (WT) and knockout (KO) mice were obtained by breeding Nrf2 heterozygote mice. All mice maintained in a 12 h light/dark cycle, pathogen-free condition. Eight-week-old Nrf2 WT and Nrf2 KO mice were randomly divided into 4 groups (*n* = 10 per group): control (Ctrl; corn oil), bixin (dissolved in corn oil), HFD-fed group (HFD), and bixin+HFD. HFD mice were generated by a high-fat diet (60 kcal% high-fat diets, D12492, Research Diets, USA) for 25 weeks. The respective control mice were fed with normal standard permitted food (D12450J) and water consumption *ad libitum*. The mice received bixin (200 mg/kg) or corn oil once every three days from the 12th week after HFD feeding. The study protocols were approved by the Soochow University Institutional Animal Care and Use Committee. Animal handling here is according to the *Guide for the Care and Use of Laboratory Animals*.

### 2.9. Hematoxylin and Eosin (H&E) and Immunohistochemistry (IHC) Staining

The tissues were embedded in the paraffin followed by fixing with 4% paraformaldehyde for 24 h. After dehydration, the slides (4 *μ*M) were cut. H&E staining was used to assess the morphologies of live tissues with the indicated treatments. The procedures of IHC and DNA damage of 8-oxo-dG were performed the same as our previous study [26]. Positive proteins' staining was performed by EnVision+System-HRP kit (Dako) based on the manufacturer's instructions. The sections were analyzed with a fluorescence microscope (Leica DM2500).

### 2.10. Biochemical Measurements

The levels of aspartate transaminase (AST), alanine aminotransferase (ALT), low-density lipoprotein (LDL) cholesterol, high-density lipoprotein (HDL) cholesterol, total cholesterol (TC), and triglyceride (TG) were measured by the respective kit according to the manufacturer's instructions. Kits of AST (C0010-2), ALT (C009-2), LDL (A113-1-1), HDL (A112-1-1), TC (A111-1), and TG (A110-1) were purchased from Nanjing Jiancheng Institute of Biotechnology (Nanjing, China).

### 2.11. Statistical Analyses

The results are expressed as the means ± SD. SPSS 19.0 was used to perform the statistical tests. Student's *t*-test (unpaired) was employed to compare multiple groups, while one-way ANOVA with Bonferroni adjustment was used to control type 1 error for multiple comparisons. A *p* < 0.05 was considered statistically significant.

## 3. Results

### 3.1. Bixin Activated the Nrf2 Signals in Liver Cells without Detectable Toxicity

**Figure 1:**
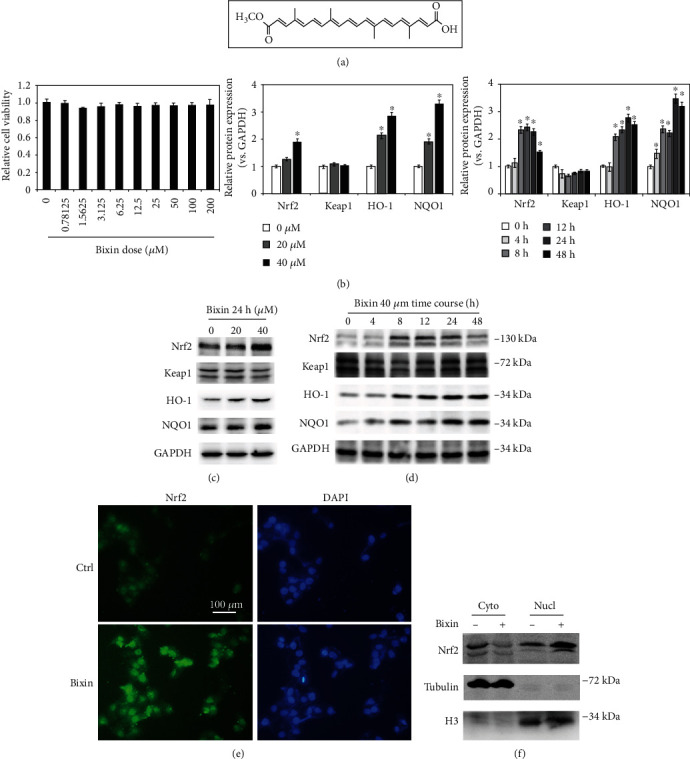
Bixin activated the Nrf2 signals in liver cells without detectable toxicity. (a) Bixin's chemical structure. (b) Cell viability was measured in LO2 cells administrated with the indicated doses of bixin for 48 h. (c) LO2 cells were administrated with bixin (0-40 *μ*M) for 24 h or treated with bixin 40 *μ*M for the indicated time (d). Cell lysates were subjected to immunoblot analyses with the indicated antibodies. Quantification of relative protein expression was determined; results are expressed as the means ± SD (^∗^*p* < 0.05, Ctrl *vs*. bixin treatments). (e) After treated with bixin (40 *μ*M) for 24 h, LO2 cells were fixed and subjected to indirect immunofluorescence staining of Nrf2 (green); nucleus was stained with DAPI (the representative images were shown, scale bar = 100 *μ*m). (f) Immunoblot analysis of Nrf2 in nuclear and cytoplasmic fractions of LO2 cells with or without bixin treatment with the indicated antibodies. Cyto: cytoplasmic fraction of LO2 cells; Nucl: nuclear fraction of LO2 cell.

According to bixin's chemical structure (Figure 1(a)) and our previous study, the cytotoxicity of bixin was first needed to be determined before investigating its effects on Nrf2 signals in LO2 cells [26]. The MTT assay showed that there was no cytotoxicity observed at the doses below 200 *μ*M (Figure 1(b)). The dose range of 0-40 *μ*M without toxicity was chosen for the subsequent studies. Immunoblot analyses indicated that bixin increased the protein expression of Nrf2, HO-1, and NQO1 in a dose-dependent manner (Figure 1(c)). And bixin (40 *μ*M) increased Nrf2 expression as early as 8 h and persisted up to 24 h. The expression of its targets (HO-1 and NQO1) was upregulated as early as 8 h and peaked at 24 h, which could persist to 48 h. Keap1, the adaptor protein of Nrf2, was a little decrease along with the upregulation of Nrf2 expression as determined in Figures 1(c) and 1(d). To verify the data, indirect immunofluorescence of Nrf2 was performed. Bixin increased the expression of Nrf2 especially in the nucleus of LO2 cells (Figure 1(e)). Then, the separation of the cytoplasm and nucleus was performed. The results showed that bixin increased the expression of Nrf2 in the nucleus (Figure 1(f)). Taken together, these data indicated that bixin could activate Nrf2 signals with no toxicity.

### 3.2. Bixin Upregulates Nrf2 Signals by Decreasing Nrf2 Ubiquitination and Increasing Its Protein Stability in a Keap1 C151-Dependent Manner

**Figure 2:**
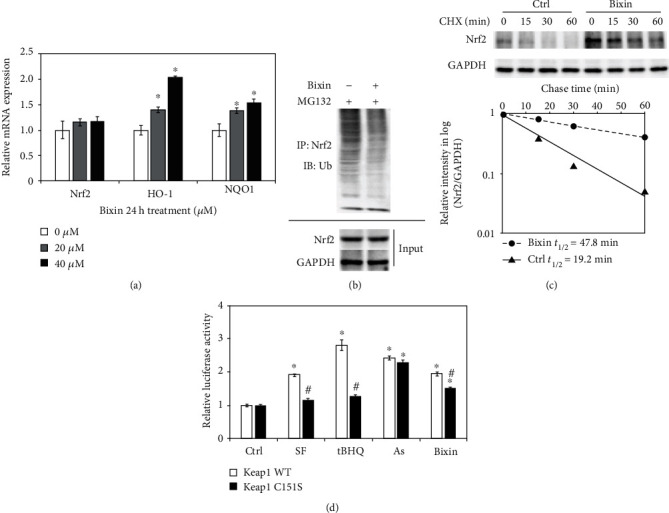
Bixin upregulates Nrf2 signals by decreasing Nrf2 ubiquitination and increasing its protein stability in a Keap1 C151-dependent manner. (a) LO2 cells were treated with the indicated doses of bixin for 24 h; the mRNA expression of Nrf2 and its targets (HO-1 and NQO1) was investigated by qRT-PCR (^∗^*p* < 0.05, Ctrl *vs*. bixin treatments). (b) LO2 cells were treated with bixin (40 *μ*M) for 24 h along with MG132 (10 *μ*M) for 4 h. Anti-Nrf2 immunoprecipitates were analyzed by immunoblot analyses with anti-Ub antibody for the detection of ubiquitin-conjugated Nrf2. (c) LO2 cells were either left untreated or treated with bixin (40 *μ*M) for 24 h. CHX (100 *μ*M) was added for the indicated time points, and cell lysates were subjected to immunoblot analyses with anti-Nrf2 and anti-GAPDH antibodies. The intensity of Nrf2 and GAPDH bands was quantified and plotted against the time after addition of CHX, and the half-life of Nrf2 was calculated. (d) LO2 cells cotransfected with the plasmids expressing either wild-type Keap1 (Keap1 WT) or C151-mutated Keap1 (Keap1 C151S) along with mGst-ARE firefly luciferase and Renilla luciferase reporters were left untreated or treated with the indicated compounds for 16 h. Dual luciferase activities were measured, and the data are expressed as the means ± SD (^∗^*p* < 0.05, Ctrl *vs*. compound-treated groups; ^#^*p* < 0.05, Keap1 WT *vs*. Keap1 C151S group).

Next, the mRNA expression of Nrf2 and its targets (HO-1 and NQO1) was investigated. As shown in Figure 2(a), bixin increased the expression of HO-1 and NQO1 at 24 h without the upregulation of Nrf2, which indicated that bixin activated Nrf2 signals on the posttranscriptional level. To further explore the mechanism of Nrf2 activation by bixin, an in vivo ubiquitination assay was performed in LO2 cells. Bixin decreased the ubiquitination of Nrf2 compared with untreated cells (Figure 2(b)). Besides that, Nrf2 protein stability also affects the activation of Nrf2 signals. Thus, the half-life of endogenous Nrf2 protein was determined in LO2 cells with bixin treatment. Bixin elongated Nrf2 half-life from 19.2 min to 47.8 min (Figure 2(c)). To further investigate whether bixin activated Nrf2 in a canonical manner, endogenous expression of Keap1 was silenced by siRNA in LO2 cells. The cells were then cotransfected with Keap1-WT or Keap1-C151S plasmids as well as ARE firefly luciferase and Renilla luciferase reporters to evaluate Nrf2 transcriptional activity. Nrf2 transcriptional activity by SF or tBHQ was inhibited with Keap1-C151S transfection (Figure 2(d)). In contrast, arsenic treatment was still able to induce Nrf2 transcriptional activity in the Keap1-C151S cells consistent with our previous finding that arsenic is a noncanonical Nrf2 inducer that works through a Keap1 C151-indenpent mechanism [18]. And bixin partially inhibited the induction of Nrf2 transcription activity in Keap1 C151S cells. Taken together, these results indicated that bixin upregulates Nrf2 signals by decreasing Nrf2 ubiquitination and increasing its protein stability, which is partially related with the critical C151 sensor residue in Keap1.

### 3.3. Bixin Activates Nrf2 by the Upregulation of P62

**Figure 3:**
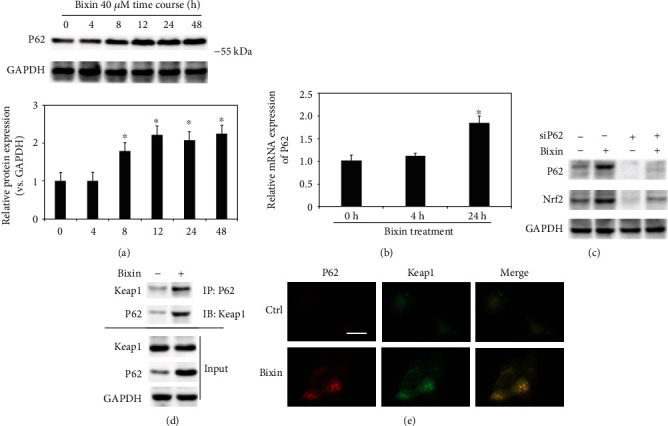
Bixin activates Nrf2 by the upregulation of P62. (a) LO2 cells were treated with bixin 40 *μ*M for the indicated time. Cell lysates were subjected to immunoblot analyses with the indicated antibodies. Quantification of relative protein expression was determined; results are expressed as the means ± SD (^∗^*p* < 0.05, Ctrl *vs*. bixin treatments). (b) LO2 cells were treated with bixin 40 *μ*M for 4 h and 24 h. mRNA was extracted, and P62 mRNA expression was detected by qRT-PCR assay; results are expressed as the means ± SD (^∗^*p* < 0.05, Ctrl *vs*. bixin treatments). (c) LO2 cells were transfected with nontargeted siRNA or P62-targeted siRNA for 24 h, and then treated with bixin 40 *μ*M for another 24 h. The indicated protein expression was investigated by immunoblot analysis. (d) LO2 cells were treated with bixin (40 *μ*M) for 24 h. Cell lysates were immunoprecipitated with the anti-P62 antibody and analyzed by immunoblot analyses with the anti-Keap1 antibody. The total protein expression was analyzed by immunoblot assay with the anti-P62, anti-Keap1, and anti-GAPDH antibodies (input). (e) LO2 cells were treated with bixin (40 *μ*M) for 24 h. The cells were fixed and subjected to indirect immunofluorescence staining of P62 (red) and Keap1 (green).

With the evidence showing that bixin activates Nrf2 signals which is partially Keap1-C151-dependent, the further mechanism of bixin on the regulation of Nrf2 was then investigated. Since Figures 3(a) and 3(b) have shown that bixin could time-dependently increase the P62 expression in mRNA and protein levels, we next silenced the expression of P62 with siRNA; the upregulation of Nrf2 by bixin was dramatically inhibited as shown in Figure 3(c). Consistent with our previous study, P62 containing a “pSTGE” domain could competitively bind with “Kelch” domain of Keap1 to activate Nrf2, and bixin could increase the expression of P62 (Figures 3(a)–3(c)). An immunoprecipitation assay was performed in LO2 cells to explore the interaction of P62 and Keap1. Bixin treatment could increase the binding of P62 and Keap1 (Figure 3(d)). Additionally, the cells were fixed and immunostained with Keap1 (green) and P62 (red). The images showed that bixin increased the colocation of Keap1 and P62 (Figure 3(e)). Taken together, these results indicated that bixin increased Nrf2 protein expression through the increased P62 competitively binding with Keap1.

### 3.4. Bixin Protects LO2 Cells from FFA-Induced Lipid Accumulation and Cytotoxicity through Nrf2

**Figure 4:**
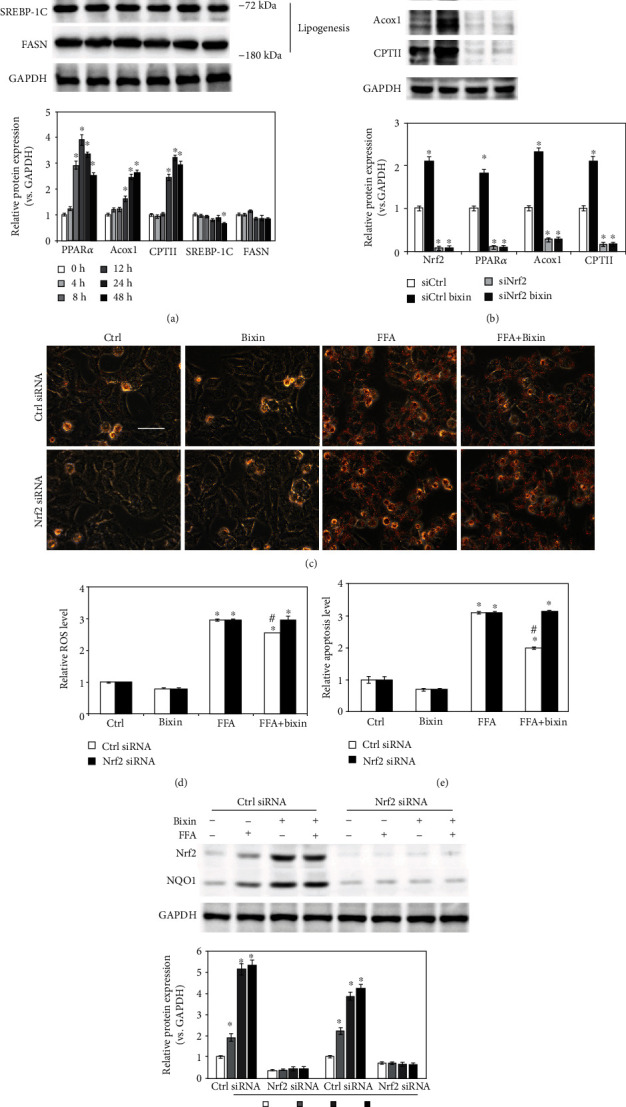
Bixin protects LO2 cells from FFA-induced lipid accumulation and cytotoxicity in an Nrf2-dependent manner. (a) LO2 cells were treated with the indicated time points of bixin (40 *μ*M). The cell lysates were subjected to the immunoblot analyses with the indicated antibodies. (b) LO2 cells were transfected with Ctrl or Nrf2 siRNA for 24 h. After pretreatment of bixin (40 *μ*M) for 24 h, the indicated protein expression was detected by immunoblot analysis. Quantification of relative protein expression was determined; results are expressed as the means ± SD (^∗^*p* < 0.05, Ctrl *vs*. treatment). (c) After transfected with siRNAs, the cells were exposed with FFA 1 mM for another 24 h cells. Cells were fixed and subjected to Oil Red O staining. The representative images from each treatment are shown (scale bar: 50 *μ*m). (d) Cells were harvested for the detection of oxidative stress caused by FFA. Harvested cells were stained with H_2_DCFA; then, the levels of ROS were detected. (e) The levels of cell apoptosis were determined. Results are expressed as the means ± SD (^∗^*p* < 0.05, Ctrl *vs*. treatment groups; ^#^*p* < 0.05, FFA group *vs*. FFA+bixin group). (f) The cell lysates were subjected to immunoblot analyses with the indicated antibodies to investigate the efficiency of Nrf2 siRNA transfection. Quantification of relative protein expression was determined; results are expressed as the means ± SD (^∗^*p* < 0.05, Ctrl *vs*. treatments).

As reported previously, Nrf2 improved the lipid accumulation caused by FFA exposure [36]. The fatty acid oxidation and lipogenesis-related proteins' expression were next evaluated with bixin treatment in our study. After cells were exposed to bixin, fatty acid oxidation-related proteins (PPAR*α* and its targets Acox1, CPTII) increased in a dose-dependent manner, but did not affect the protein expression related with lipogenesis (Figure 4(a)). In Nrf2 knockdown cells, the expression of PPAR*α* and its targets (Acox1, CPTII) was mostly inhibited as shown in Figure 4(b), which indicated that bixin upregulated the fatty acid oxidation-related proteins in the Nrf2-dependent manner. Next, the prevention of lipid accumulation was investigated in the cells exposed with FFA. The results from Oil Red O staining showed that the lipid accumulation was dramatically decreased in bixin-treated cells, while siNrf2 could attenuate this prevention (Figure 4(c)). Then, the feasibility of bixin for cytoprotection against FFA-induced toxicity was investigated. Oxidative stress and cell apoptosis were measured after LO2 cells were challenged by FFA with or without bixin pretreatment. Bixin pretreatment decreased the levels of ROS and cell apoptosis induced by FFA exposure. Bixin alone has no effects, which indicated that bixin itself could not trigger the oxidative stress and has less toxicity. However, bixin-mediated suppression was not detected in Nrf2 knockdown cells, indicating that the cytoprotection of bixin was through Nrf2 signals (Figures 4(d) and 4(e)). These data indicated that bixin could attenuate the lipid accumulation, maintain the redox balance upon FFA exposure, and protect cells from FFA-caused cell apoptosis through Nrf2 signals.

### 3.5. Bixin Blunts Hepatosteatosis and Reduces Circulating Lipid Levels in HFD-Fed Mice through Nrf2 Signals

**Figure 5:**
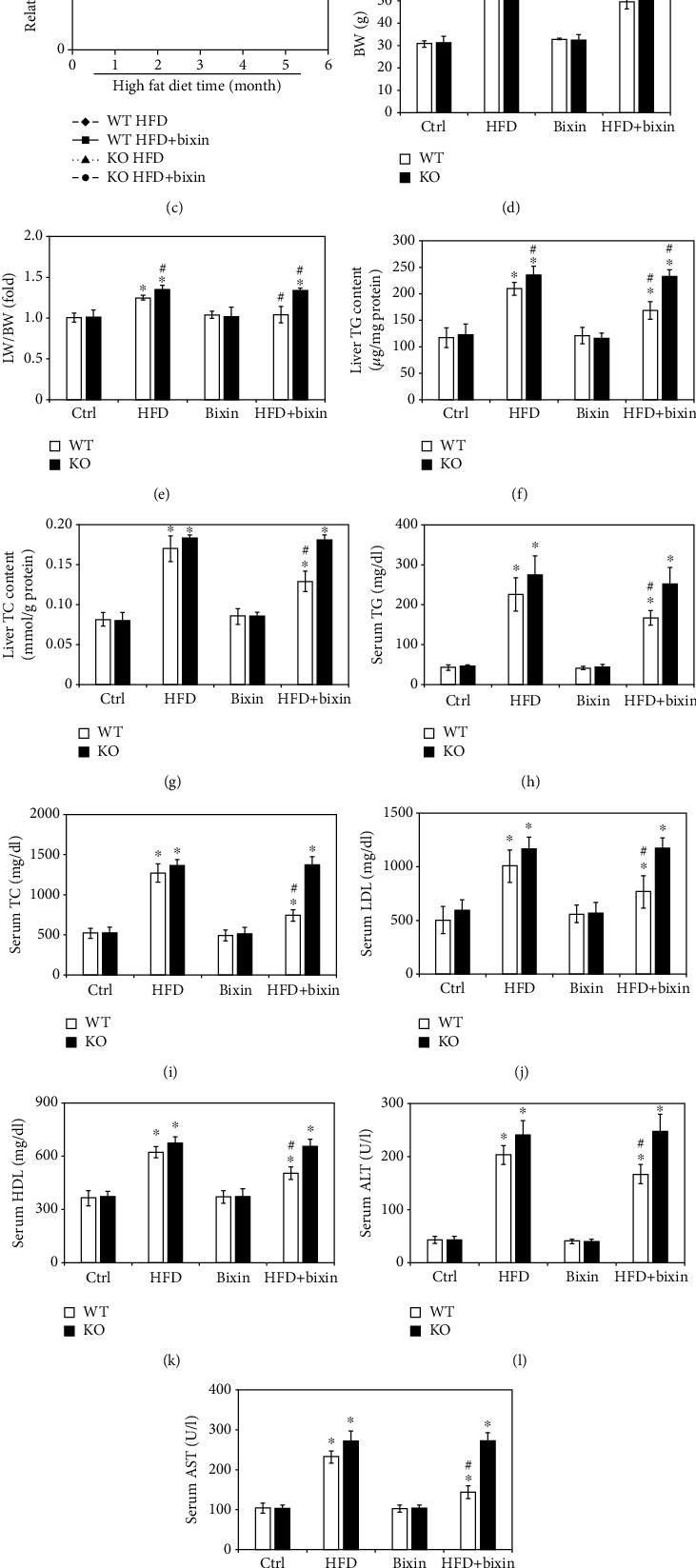
Bixin blunts hepatosteatosis and reduces circulating lipid levels in HFD-fed mice through Nrf2 signals. (a) Schematic representation of HFD feeding and bixin administration duration in Nrf2 WT and KO mice. (b) Food consumption, (c) water consumption, (d) BW, (e) LW/BW, (f) liver TG content, (g) liver TC content, (h) serum TG, (i) serum TC, (j) serum LDL, (k) serum HDL, (l) serum ALT, and (m) serum AST of mice with the indicated treatments are provided. Results are expressed as the means ± SD (*n* = 10; ^∗^*p* < 0.05, Ctrl *vs*. treatment groups; ^#^*p* < 0.05, HFD group *vs*. HFD+bixin group). (n) Liver tissue sections from different groups of Nrf2 WT and Nrf2 KO mice were subjected to Oil Red O staining (*n* = 10; scale bar = 100 *μ*m. Representative images from each group are shown).

To explore the impacts of bixin on the regulation of Nrf2 signals in the process of hepatic lipid metabolism, Nrf2 WT and KO mice with a long-term HFD were employed in this study. The duration of HFD feeding and bixin administration is shown in Figure 5(a). There is no significant difference of food and water consumption among the indicated group of mice (Figures 5(b) and 5(c)). After 25 weeks on the HFD, body weight (BW), the ratio of liver weight to BW (LW/BW), liver TG contents, and liver TC contents were significantly increased, which was inhibited by bixin in Nrf2 WT mice (Figures 5(d)–5(g)). And Nrf2 KO mice exhibited aggravated hepatic steatosis in comparison with HFD-fed mice, showing increased levels of BW, LW/BW, and liver TG contents, which could not be decreased by bixin administration. In addition, the circulating lipid levels were also investigated. After 25 weeks on the HFD, both Nrf2 WT and KO mice developed marker hypercholesterolemia. Serum total cholesterol (TC), serum low-density lipoprotein (LDL), and serum high-density lipoprotein (HDL) cholesterol levels showed a little increase in Nrf2 KO mice without significant difference (Figures 5(f)–5(k)). Bixin could reduce the levels of serum TC, serum LDL, and serum HDL only in Nrf2 WT mice as well. In addition, the hepatic functional changes were measured accordingly; serum aminotransferase (ALT) and aspartate aminotransferase (AST) were determined. HFD feeding increased the levels of ALT and AST, which were blunted by bixin administration (Figures 5(l) and 5(m)). Oil Red O staining exhibited widespread lipid vacuoles deposited in the liver sections of both Nrf2 WT and KO mice with HFD. And bixin administration could only decrease the lipid accumulation in the livers of Nrf2 WT mice (Figure 5(n)). Taken together, these results demonstrated that bixin could reduce the lipid accumulation and improve liver dysfunction in the Nrf2-dependent manner.

### 3.6. Bixin Upregulates Nrf2 Signals and PPAR*α* Involved in HFD-Induced Hepatic Steatosis

**Figure 6:**
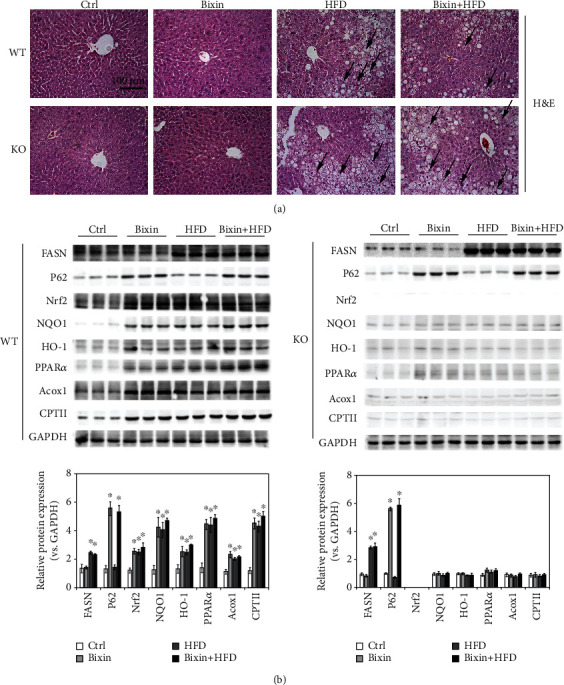
Bixin upregulates Nrf2 signals and PPAR*α* involved in HFD-induced hepatic steatosis. (a) Liver tissue sections from different groups of Nrf2 WT and Nrf2 KO mice were subjected to Oil Red O staining; black arrow indicates the infiltration of inflammatory cells. (*n* = 10; scale bar = 100 *μ*m. Representative images from each group are shown). (b) Liver tissue lysates from each group were subjected to immunoblot analyses with the indicated antibodies. Representative blots of three independent samples from each group were shown; quantification of relative protein expression was determined; results are expressed as the means ± SD (*n* = 10; ^∗^*p* < 0.05, Ctrl *vs*. treatment groups; ^#^*p* < 0.05, HFD group *vs*. HFD+bixin group).

To further explore the feasibility of Nrf2 signals regulated by bixin in prevention against HFD-induced hepatic steatosis, H&E staining was then performed to investigate the tissue pathology. Consistent with the Oil Red O staining, H&E staining revealed cell ballooning in the liver of HFD-fed mice in both Nrf2 WT and KO mice. In addition, Nrf2 deficiency aggravated the lipid accumulation in the liver tissue section, which is consistent with Figures 5(e) and 5(f). Bixin injection did not cause any pathological changes of the tissue. Importantly, in Nrf2 WT mice fed with HFD and cotreated with bixin, hepatic pathological changes were ameliorated (Figure 6(a)). Then, the related protein expression was next detected (Figure 6(b)). HFD induced the expression of fatty acid synthase (FASN), an indicator of liver steatosis in both Nrf2 WT and KO mice. And the expression of FASN is higher in the liver of Nrf2 KO mice as well. Besides that, the expression of P62 was increased with bixin treatment in both Nrf2 WT and KO mice. The protein expression of Nrf2 and its targets (NQO1, HO-1) increased in the liver tissue of Nrf2 WT mice with bixin injection, HFD feeding, or combination compared with the control group. Then, the expression of PPAR*α* and its targets (Acox1, CPTII) was explored in the liver sections of both Nrf2 WT and KO mice. Consistent with the in vitro study, bixin could increase the expression of PPAR*α* and its targets via Nrf2. In Nrf2 WT mice, the expression of PPAR*α* and its targets (Acox1, CPTII) increased in the bixin, HFD, and combination groups of mice. And there is no significant induction of lipid metabolism-related protein (PPAR*α* and its targets) in the liver with Nrf2 deficiency. Interestingly, it was also reported that using combined supplementations such as DHA and HT is warranted to prevent liver steatosis through regulating PPAR*α* and Nrf2 signals. Given this, these results indicated that bixin upregulates Nrf2 signals and PPAR*α* involved in HFD-induced hepatic steatosis.

### 3.7. Bixin Attenuates the Hepatic Inflammation and Blunts the Liver Abnormality through Nrf2

**Figure 7:**
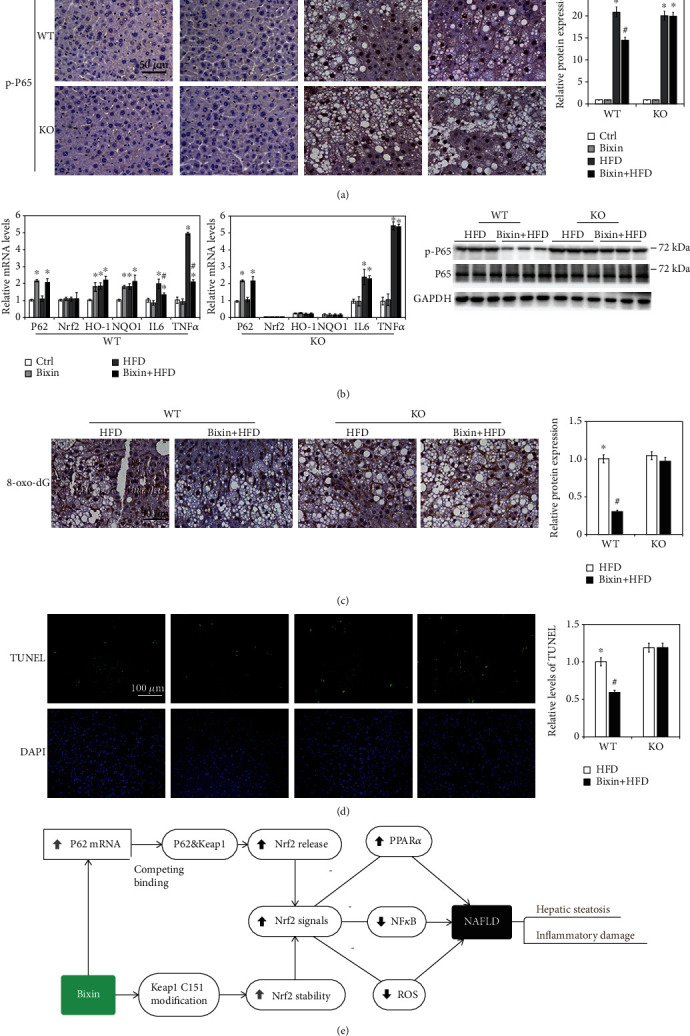
Bixin attenuates the hepatic inflammation and blunts the liver abnormality through Nrf2. (a) Liver tissue sections from each group of mice were subjected to IHC staining of p-P65. (b) The total RNA from different group was extracted. mRNA levels of P62, Nrf2, HO-1, NQO1, IL-6, and TNF-*α* were measured by qRT-PCR assay; results are expressed as the means ± SD (^∗^*p* < 0.05, Ctrl *vs*. treatment groups; ^#^*p* < 0.05, FFA group *vs*. FFA+bixin group). (c) The IHC staining of 8-oxo-dG in the liver tissue from the HFD and bixin+HFD groups of Nrf2 WT and KO mice was performed. (d) TUNEL staining of liver tissue from the HFD and bixin+HFD groups of Nrf2 WT and KO mice (DAPI indicates the nucleus in liver tissues). Representative images from each group are shown (*n* = 6; scale bar = 50 or 100 *μ*m; ^∗^*p* < 0.05, Ctrl *vs*. treatment groups; ^#^*p* < 0.05, HFD group *vs*. HFD+bixin group). (e) Proposed model for the therapeutic action of bixin against NAFLD: bixin administration could activate Nrf2 signals through two different mechanisms: (i) canonical mechanism, which modifies the critical cysteine residues in Keap1, leading to a conformational change of Keap1-Cul3-E3 complex that releases the bind with DLG motif of Nrf2 and subsequently stabilized Nrf2 and (ii) noncanonical mechanism that P62 binds with the Kelch domain of Keap1 with its pSTGE motif to stabilize Nrf2. The upregulation of Nrf2 improves the hepatic steatosis and inflammatory damage.

H&E staining exhibited the HFD-caused liver steatosis combined with the infiltration of inflammation cells. NF-*κ*B is the most common signal regulating inflammatory response, and the protein levels of p-P65 were employed to assess the activation of NF-*κ*B. A significant increase of p-P65 protein was observed with long-term HFD in both Nrf2 WT and KO mice. Bixin alone did not affect either p-P65 or P65 expression. In the HFD-fed Nrf2 WT mice injected with bixin, p-P65 protein levels were significantly decreased compared to those in HFD-fed mice (Figure 7(a)). And the qRT-PCR analysis of interleukin- (IL-) 6 and TNF-*α* confirmed that HFD-fed activated NF-*κ*B in both types of mice and bixin attenuated this activation only in Nrf2 WT mice (Figure 7(b)). Next, IHC analysis for 8-hydroxy-2′-deoxyguanosine (8-oxo-dG) was performed to detect HFD-caused oxidative DNA damage. HFD dramatically enhanced 8-oxo-dG staining in both types of mice, indicating the serious oxidative stress in the tissue, which could adoptively induce Nrf2 signals. Bixin itself did not have any effect, but could suppress 8-oxo-dG staining caused by HFD in Nrf2 WT mice (Figure 7(c) and S1). To further confirm that, the cell apoptosis was investigated by TUNEL assay. HFD-fed-caused cell apoptosis could be repressed in only Nrf2 WT mice as showed in Figure 7(d) and S1. Taken together, these results indicated that bixin attenuates the hepatic inflammation and blunts the liver abnormality in the Nrf2-dependent manner.

## 4. Discussion

NAFLD is a common liver disease worldwide. Hepatic steatosis and inflammation are the main characteristics of NAFLD, which also affect the prognosis as well [7, 37]. An urgent need exists for the exploration of efficient strategies that prevent and limit lipid accumulation and inflammatory response in the duration of NAFLD. Bixin, extracted from the seeds of Bixa orellana, has emerged as defined antioxidant and anti-inflammatory properties, which could prevent oxidative DNA damage and lipid peroxidation. And in our previous study, we identified bixin is a novel Nrf2 activator, which could suppress the NF-*κ*B regulated inflammatory response and improve the lung fibrosis through inhibition of TGF*β*1 signals in an Nrf2-dependent manner [22, 26]. Nrf2 serves as an important role against inflammation and has been confirmed to suppress HFD-induced oxidative stress with hydroxytyrosol administration. And according to the previous studies, AR-EVOO supplementation reversed IRD-induced oxidative stress, lipid peroxidation, and protein oxidation through NF-*κ*B inactivation. Based on this, here we mainly focused on the mechanism of Nrf2 signals' activation by bixin and explored its potential feasibilities in HFD-caused hepatic steatosis and inflammatory response.

In this study, we demonstrated that bixin activates Nrf2 signals at the posttranscriptional level by decreasing the Nrf2 ubiquitination and stabilizing its protein expression (Figure 1). We found that the induction of Nrf2 signals was partially related with the critical C151 sensor residue in Keap1 (Figure 2). In addition, bixin could also enhance the protein and mRNA expression of P62, which could interact with Keap1 “Klech” domain through its “STGE” motif (Figure 3). Thus, bixin administration could activate Nrf2 signals through two different mechanisms: (i) canonical mechanism, which modifies the critical cysteine residues in Keap1, leading to a conformational change of Keap1-Cul3-E3 complex that releases the bind with DLG motif of Nrf2 and subsequently stabilized Nrf2 [32] and (ii) noncanonical mechanism that P62 binds with the Kelch domain of Keap1 with its pSTGE motif to stabilize Nrf2 [33].

The induction of Nrf2 signals inhibited the NF-*κ*B signaling pathway and decreased the oxidative DNA damage, which attenuated inflammatory response, cell apoptosis, and tissue functional disorder and histological damage (Figures 4–7). In addition, according to the previous reports, Nrf2 signals activated PPAR*α* signals, which can restrain lipid peroxidation and attenuate FFA accumulation in the tissues [36]. Together with the report that bixin protects against lipid peroxidation, the expression of PPAR*α* and its targets (Acox1 and CPTII) was investigated (Figures 4(a) and 4(b)) [38]. We found that PPAR*α* and its targets were upregulated by bixin in a dose- and time-dependent manner (Figure 4(a)). As a result, bixin decreased the accumulation of lipid in the cells and liver tissues as well, while bixin could not improve the situation in the cells with Nrf2-siRNA transfection, which indicated that bixin regulated the PPAR*α* signals and suppressed the lipid accumulation via Nrf2 (Figures 4(c) and 6(b)). Our next step of study will directly focus on the regulation of Nrf2 and PPAR*α* signals by bixin and its role in the lipid metabolism.

Then, in this work, we also unveiled the mechanism of P62 induced by bixin on the induction of Nrf2 signals. We found that bixin treatment increased the level of P62 (Figures 3(a) and 3(b)). According to the previous studies, there is a “STGE” domain in the protein of P62, which could interact with the Kelch domain of Keap1, leading to the release of Nrf2 [39–41]. Therefore, we analyzed the interaction of P62 and Keap1 by immunoprecipitation and immunofluorescence (Figures 3(d) and 3(e)). After bixin treatment, the binding of P62 and Keap1 was increased as data shown in Figure 4(d), which indicated that bixin activated Nrf2 by increasing P62 to connect with Keap1. And silencing the P62 expression, which reduced this interaction, could affect the increase of Nrf2 caused by bixin (Figure 4(c)). In addition, as demonstrated previously, there are two different mechanisms of Nrf2 induction [32, 34, 42]. ROS and most of classical Nrf2 inducers activate Nrf2 by modification of critical cysteine residues in Keap1, which usually causes the obvious cytotoxicity even though Nrf2 expression can be dramatically increased accordingly, such as the upregulation of Nrf2 caused by HFD in Figure 6(b) [43]. Here except for the canonical mechanism, bixin activated Nrf2 via the induction of P62, which may be contributed to its low toxicity characteristic. In addition, compared with single treatment, the Nrf2 signaling pathway did not show further increase in the liver of mice from the combination group. The reason for this may be because bixin pretreatment could activate Nrf2 signals, which upregulate the antioxidant capability in the tissue and directly polish the accumulative lipid-caused ROS and protect the tissue cells from the oxidative stress damage.

Cumulative studies have reported that NAFLD is the hepatic manifestation of metabolic syndrome [5, 44]. For example, the hepatic steatosis and inflammation could be caused by the abnormal lipid metabolism and chronic inflammation in the adipose tissue in the metabolic diseases, which means that the hepatic disorder would be solved by etiological correction [45, 46]. But as we have known, these kinds of diseases are usually complicated with multiple factors for their initiation and development, which are difficult to target and treat with. Bixin here was conferred with the cytoprotection to the liver tissue against lipid accumulation and inflammation induced by HFD-fed mice, which is important for the tissues that have already been injured. Bixin i.p. injection could also increase the antioxidant and inflammatory capability of other tissues in HFD-caused metabolic syndrome, which may cooperatively improve these hepatic abnormalities as well. Bixin-based Nrf2-directed systemic intervention may also provide therapeutic benefit in protecting other organs in the process of metabolic syndrome.

## 5. Conclusion

Bixin activated the Nrf2 signals through both canonical and noncanonical mechanisms and represents a prototype Nrf2 activator that displays cytoprotective activity upon system administration targeting hepatic steatosis and oxidant inflammation originating from long-term HFD-fed mice.

## Data Availability

The data used to support the findings of this study are available from the corresponding author upon request.

## Abbreviations

ALT: Alanine aminotransferase
AST: Aspartate transaminase
BW: Body weight
CA: Cinnamaldehyde
CHX: Cycloheximide
Cp: Crossing point
DMEM: Dulbecco's modified Eagle's medium
FATPs: Fatty acid transport proteins
FDA: Food and Drug Administration
HDL: High-density lipoprotein
H&E: Hematoxylin and eosin
HFD: High-fat diet
HRP: Horseradish peroxidase
IHC: Immunohistochemistry
Keap1: Kelch-like ECH-associated protein 1
KO: Knockout
LDL: Low-density lipoprotein
IL-6: Interleukin-6
NAFLD: Nonalcoholic fatty liver disease
NASH: Nonalcoholic steatohepatitis
Nrf2: NF-E2 p45-related factor 2
PPAR*α*: Peroxisome proliferator-activated receptor *α*
ROS: Reactive oxygen species
SD: Standard deviations
SF: Sulforaphane
T-I: Tanshinone I
TC: Total cholesterol
TG: Triglyceride
WT: Wild-type
8-oxo-dG: 8-Hydroxy-2′-deoxyguanosine.

## Supplementary Materials

Supplementary Materials Supplementary Figure 1: the IHC staining of 8-oxo-dG and TUNEL staining of liver tissue from the Ctrl and bixin groups of Nrf2 WT and KO mice (DAPI indicates the nuclear in the liver tissues; *n* = 6; scale bar = 100 *μ*m).
