# Supplementary figures and images for: Bixin Attenuates High-Fat Diet-Caused Liver Steatosis and Inflammatory Injury through Nrf2/PPARα Signals
Source: Oxid Med Cell Longev. 2021 Feb 2;2021:6610124. doi: 10.1155/2021/6610124 (PMC7872754; doi:10.1155/2021/6610124)

**S1**

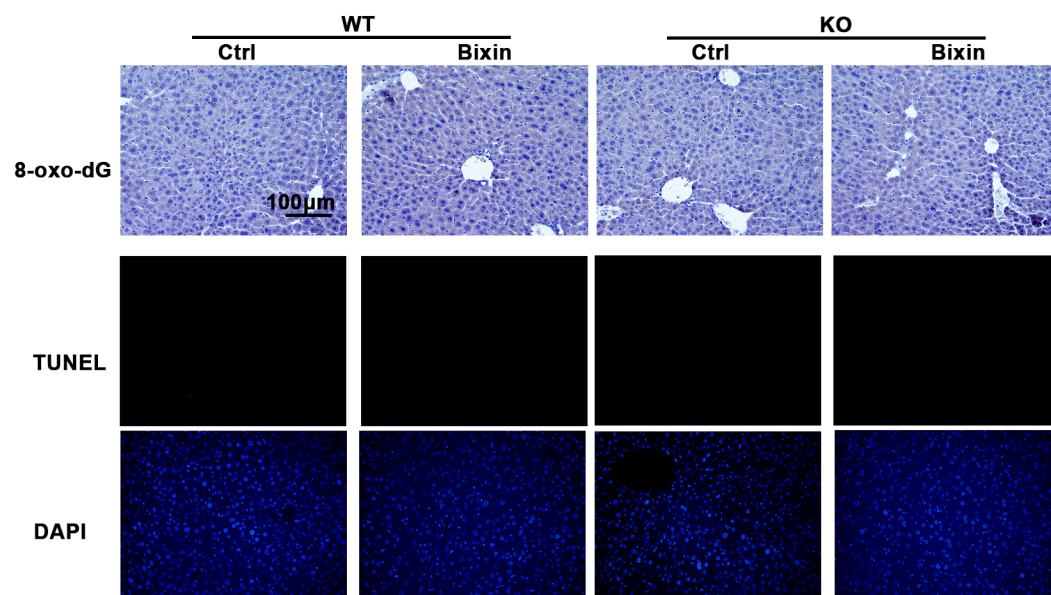

Supplement: Supplementary Materials — Supplementary Figure 1: the IHC staining of 8-oxo-dG and TUNEL staining of liver tissue from the Ctrl and bixin groups of Nrf2 WT and KO mice (DAPI indicates the nuclear in the liver tissues; n = 6; scale bar = 100 μm). [file 6610124.f1.pdf]
